# Supplementary material for: Humanized Monoclonal Antibody Against Citrullinated Histone H3 Attenuates Myocardial Injury and Prevents Heart Failure in Rodent Models
Source: Biomolecules. 2025 Aug 20;15(8):1196. doi: 10.3390/biom15081196 (PMC12384098; doi:10.3390/biom15081196)
Supplement: Supplementary file 1 [file biomolecules-15-01196-s001.zip › biomolecules-3763298-supplementary.pdf]

Humanized Monoclonal Antibody Against Citrullinated Histone H3 Attenuates Myocardial Injury and Prevents Heart Failure in Rodent Models

Uncropped images with densitometry readings/intensity ratios

Fig 1C

Fig 1C

IL-1β (ab9722)

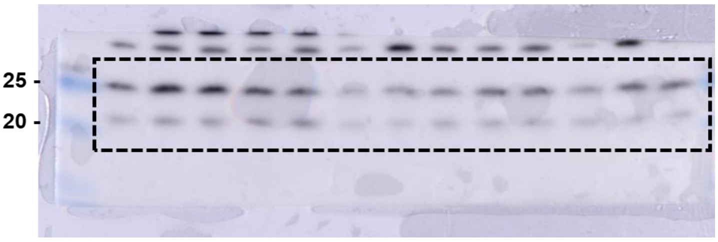

	Density		
Mice	20kD	25kD	Total
#1	3031.309	8881.288	11912.6
#2	5637.744	15566.84	21204.59
#3	5536.087	13645.14	19181.22
#4	6162.078	10965.95	17128.02
#5	6599.421	9867.045	16466.47
#6	1893.539	3846.246	5739.785
#7	1767.731	5090.631	6858.362
#8	3025.702	5727.631	8753.333
#9	3040.066	7640.945	10681.01
#10	3109.986	7818.48	10928.47
#11	2568.016	5363.56	7931.576
#12	2549.037	8605.702	11154.74
#13	2526.874	7569.409	10096.28

Fig 2A

Fig 2A

CS (ab129095)

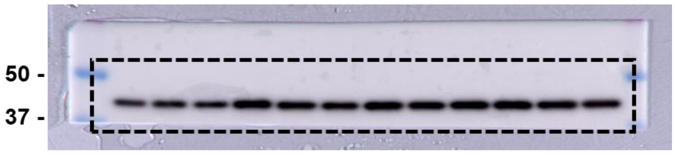

GAPDH (ab8245)

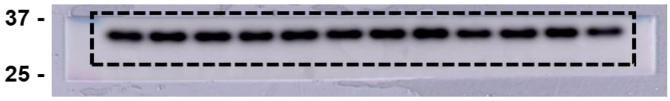

Mice	CS	GAPDH	Ratio
#1	13927.07	27382.79	0.508606
#2	16889.38	29929.14	0.564312
#3	15123.77	29906.02	0.50571
#4	27508.04	28436.72	0.967342
#5	23248.67	30075.02	0.773023
#6	22858.26	28947.14	0.789655
#7	26332.14	30789.43	0.855233
#8	24960.43	32926.97	0.758054
#9	26992.33	26829.02	1.006087
#10	26833.79	28409.84	0.944525
#11	26638.79	31082.38	0.857038
#12	23932.67	25595.26	0.935043

Fig 1D

Fig 1D

IFN-β (27506-1-ap)

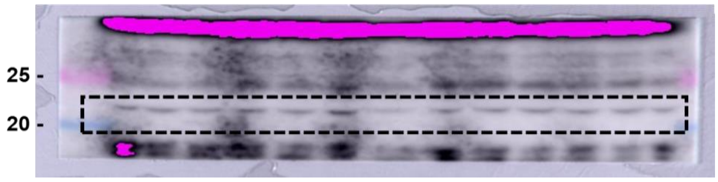

Mice	Density 22kD
#1	4097.61
#2	3804.095
#3	3850.832
#4	5031.53
#5	3860.246
#6	6049.459
#7	1529.518
#8	3794.711
#9	3438.489
#10	3316.368
#11	3225.706
#12	3188.882
#13	2585.104

Fig 2B

Fig 2B

CS (ab129095)

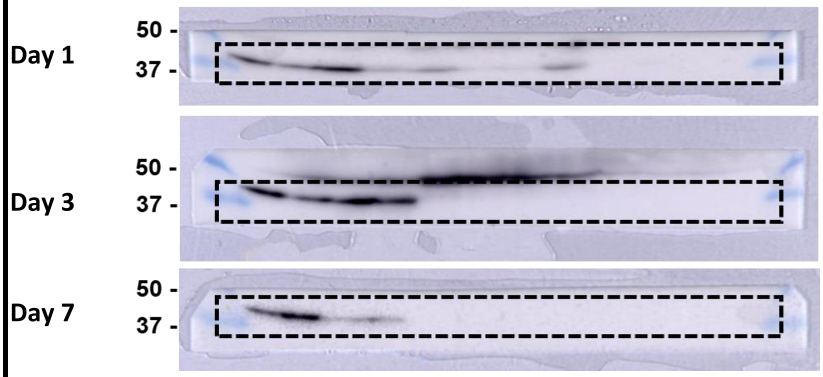

	Mice	Density Day1	Density Day3	Density Day7
Control	#1	12420.6	15049.92	9534.631
Control	#2	8999.782	10382.58	14443.35
Control	#3	13799.07	19197.69	4774.451
Control	#4	4408.158	15611.27	5023.765
Treated	#5	6861.158	451.92	454.406
Treated	#6	1057.054	460.92	452.456
Treated	#7	1033.163	482.749	454.113
Treated	#8	7990.128	523.678	456.062
Sham	#9	452.406	432.113	454.406
Sham	#10	484.335	449.971	452.042
Sham	#11	488.607	450.95	454.406
Sham	#12	496.192	460.75	452.071

Fig 4A

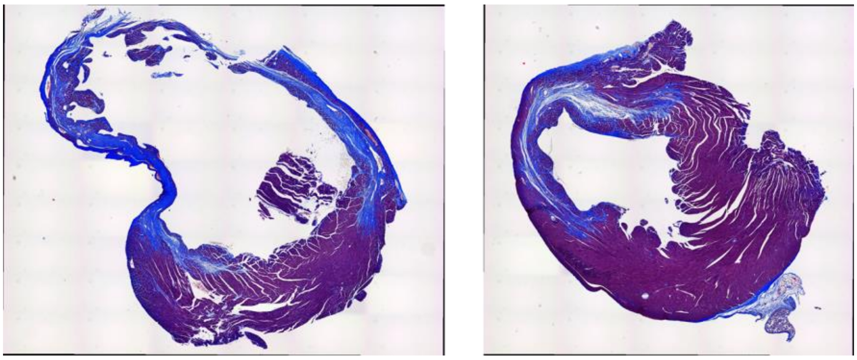

Fig 3A

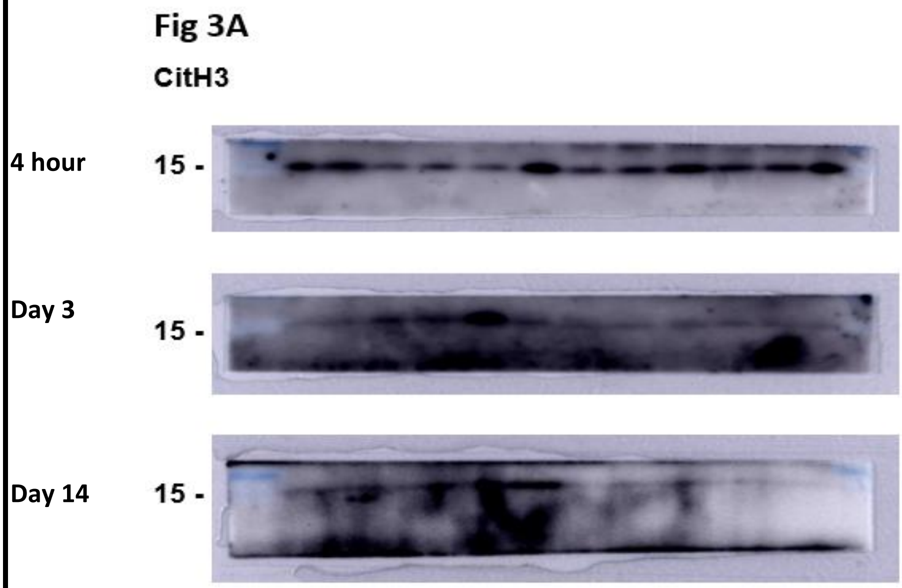

	Density		
Mice	4 hours	Day 3	Day 14
#1	35392.22	3945.175	7023.513
#2	41936.92	5430.602	12090.64
#3	15316.77	18880.66	4783.593
#4	15209.69	25648.74	10984.37
#5	14292.04	41504.07	22940.69
#6	39908.27	12935.42	20081.41
#7	30553.23	8258.886	1028.241
#8	33681.34	5288.702	3995.229
#9	38967.29	7071.702	6748.317
#10	17239.48	5429.882	1012.38
#11	27784.99	4155.184	2381.288
#12	36541.12	4611.468	1014.054
